# Caryophyllene Oxide, the Active Compound Isolated from Leaves of *Hymenaea courbaril* L. (Fabaceae) with Antiproliferative and Apoptotic Effects on PC-3 Androgen-Independent Prostate Cancer Cell Line

**DOI:** 10.3390/molecules26206142

**Published:** 2021-10-12

**Authors:** Claudia Delgado, Gina Mendez-Callejas, Crispin Celis

**Affiliations:** 1 Phytochemistry Research Group (GIFUJ), Department of Chemistry, Faculty of Science, Javeriana University, 7th Street # 40-62, Bogotá 110231, Colombia; delgado.claudia@javeriana.edu.co; 2 Group of Biomedical Research and Applied Human Genetics (GIBGA), Laboratory of Cellular and Molecular Biology, School of Medicine, University of Applied and Environmental Sciences (U.D.C.A), 222 Street # 55-37, Bogotá 111166, Colombia

**Keywords:** *Hymenaea courbaril* L., apoptotic proteins, caryophyllene oxide, cytotoxicity, PC-3 prostate cancer cells

## Abstract

Cancer treatment frequently carries side effects, therefore, the search for new selective and effective molecules is indispensable. *Hymenaea courbaril* L. has been used in traditional medicine in South America to treat several diseases, including prostate cancer. Leaves’ extracts from different polarities were evaluated using the 3-(4,5-methyl-thiazol-2-yl)-2,5-diphenyl-tetrazolium bromide (MTT) cell viability assay to determine the cytotoxicity in prostate p53-null cells, followed by bio-guided fractionations to obtain the most cytotoxic fraction considering the selectivity index. The most cytotoxic fraction was analyzed by GC/MS to identify the active compounds. The majority compound, caryophyllene oxide, induced early and late apoptosis, depolarized the mitochondrial membrane, leading to several morphological changes and shifts in apoptotic proteins, and caspases were evidenced. Depolarization of the mitochondrial membrane releases the pro-apoptotic protein Bax from Bcl-xL. The apoptosis process is caspase-7 activation-dependent. Caryophyllene oxide is a safe anti-proliferative agent against PC-3 cells, inducing apoptosis with low toxicity towards normal cells.

## 1. Introduction

Prostate cancer (PC) has become a leading cause of cancer-related death in men all over the world. Most PCs are usually acinar adenocarcinomas characterized by glandular formation, absence of basal cells, uncontrolled proliferation of tumor cells, and expression of differentiation luminal markers, such as the androgen receptor (AR) and the prostate-specific antigen (PSA), and the majority of these adenocarcinomas are androgen-dependent, which allows hormone therapy that inhibits AR signaling [1,2,3,4]. There are variant forms of prostatic epithelial malignancies, such as the Small-Cell Neuroendocrine Carcinoma (SCNC), which are rare tumors that account for no more than 1% of all prostate carcinomas [5,6]. They are often seen as recurrent tumors in patients who have received hormone therapy and become resistant to hormone-suppressive therapy [2,4,5]. The normal prostate epithelium is constituted by luminal epithelial cells, basal cells, and a minor component of neuroendocrine (NE) cells that are scattered throughout the prostate [1,6]. SCNC tumors can be found as a pure form of neuroendocrine cells or as a component of mixed tumors. NE tumor cells are androgen-independent and do not express AR, therefore, they do not respond to therapies directed to the AR pathway. Generally, SCNC is considered a lethal disease without effective treatment options other than chemotherapy [2,4].

PC-3 is a characteristic cell line of SCNC since it shares many traits, such as the expression of NE markers such as Chromogranin A (CgA) and neuronal-specific enolase (NSE), the absence of expression of the differentiation luminal markers, AR and PSA, and the p53 mutation (TP53 hemizygotes for chromosome 17), where the single copy of the p53 gene has a base deletion in codon 138 (GCC/GC) [7]. It causes negative p53-dependent apoptosis, though in this way, p53 not only loses its tumor suppressor function but also acquires new oncogenic properties, which promote cell growth, inhibit cell death, and increase drug resistance [1,2,3].

High mortality rate, systemic toxicity, multidrug resistance, excessive sensitivity to conventional therapies, and the lack of specificity of synthetic drugs that display lethal effects on normal cells limit the successful results of conventional therapy, decreasing the patient’s life quality [8]. Ideally, cancer therapies should have selective effects on cell destruction, requiring an urgent search for effective, targeted, and safer drugs [5]. Historically, natural products have been used since ancient times in folk medicine, with plants being a common alternative for cancer treatment in many countries, especially developing countries. Secondary metabolites obtained from natural products and their synthetic derivatives can be considered as a potential source of new compounds for the treatment of cancer [9]. 

*Hymenaea courbaril Linneo* (Sp. PI. 1192. 1753) is commonly known as West Indian Locust, Algarroba, Amami-gum, Brazilian copal, Courbaril, Farinheira, Guapinol, Imbuiva, Jataiba, Jatoba-da-catinga, Jatoba-miudo, Jitai, Kaurubali, Paquio, Simiri, and Stinking toe (Plants for a Future, n.d.), among other names, and can vary and be unique between different countries. It is a neotropical plant belonging to the Fabaceae family. The West Indian Locust has a long history of use by the indigenous tribes of the rainforest and in traditional South American medicine, used in different ways. Various parts of the plant, such as bark, fruits, resin, leaves, seeds, stems, and vapors of oleoresin, have been associated with its ethnomedicinal properties. Forest communities use their parts in decoction, infusion, tincture, poultices, juice, soaking, or licking for the treatment of various diseases, such as respiratory distress, stomachache, wounds, fractures, anemia, prostatic ailments, and some types of cancer, such as leukemia [10,11,12] and prostate cancer, among others [13].

Studies related to the *H. courbaril* plant involve the cytotoxic activity of the extracts, but there are not enough data available in the literature on the induction of apoptosis from extracts, fractions, or their metabolites. This study aimed to evaluate the antiproliferative effect of extracts and fractions, and the apoptotic effect of the major secondary metabolite from the leaves of the plant *Hymenaea courbaril* in androgen-independent prostate cancer cells, PC-3, and contribute to its ethnobotanical knowledge.

## 2. Results and Discussion

### 2.1. Hexane Extract Bio-Guided Fractionation Led to Active Fractions on PC-3 and MRC-5 Cells

**Figure 1:**
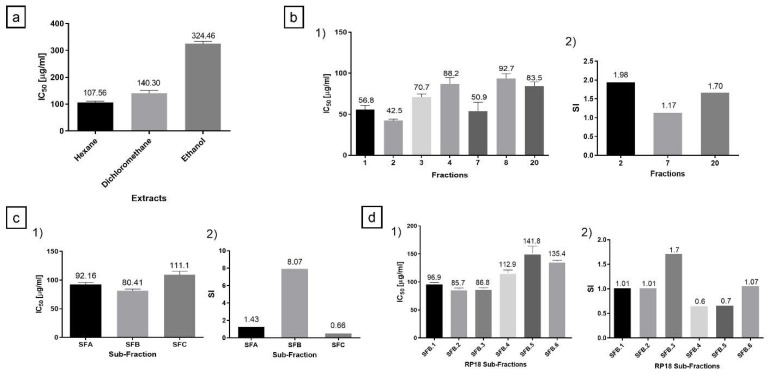
Cytotoxic activity of the extracts, fractions, and subfractions. (**a**) IC_50_ of the hexane, dichloromethane, and ethanol total extracts obtained from *H. courbaril* leaves in the PC-3 tumor cell line. (**b**(1)) Cytotoxic activity of the seven fractions with the highest activity on the PC-3 cell line. (**b**(2)) Selectivity Index of the fractions. (**c**(1)) The IC_50_ value of the sub-fractions of the fraction-2 with the highest activity on the PC-3 cell line. (**c**(2)) Selectivity Index of the sub-fractions of the fraction-2. Somehow, throughout fraction-2 fractionation, the cytotoxic activity was diminished. It changed from IC_50_ = 42.53 (fraction-2) to IC_50_ = 80.41 µg/mL (SFB). (**d**(1)) The cytotoxic activity of the six sub-fractions obtained in the RP18 reverse-phase chromatography from the SFB sub-fraction. (**d**(2)) Selectivity Index of these six sub-fractions obtained in the reverse-phase chromatography. SFB.3 sub-fraction presented the highest anti-cancer potential, with a moderately cytotoxic IC_50_ of 86.81 µg/mL and a SI of 1.71. The experiments were carried out in triplicate with two replicas.

Total extracts were evaluated by the MTT assay in PC-3 cells. The hexane extract had the highest cytotoxic activity with an IC_50_ of 107.56 μg/mL (Figure 1a). According to the suggested criteria for cytotoxic activity in vitro, total extracts and fractions are considered moderately cytotoxic when the IC_50_ ranges between 21 and 200 µg/mL [14,15]. Hence, it was considered moderately cytotoxic, and thereby was selected to continue with the bio-guided fractionation. Of the hexane extract, 10 g was fractionated in 320 g of silica gel-60 on column chromatography with an isocratic phase of petroleum ether–acetone 8:2. Twenty fractions were obtained after the MTT assay was performed, in which seven fractions were identified as active, with IC_50_ ranging between 42.53 and 92.72 μg/mL (Figure 1b(1)). To discriminate between the active fractions and continue with the bio-guided process, the Selectivity Index (SI) was assessed using a tumor (PC-3) and a normal cell line (MRC-5), where a higher value is indicative of protection to a normal cell against increased toxicity towards the tumor cell (Figure 1b(2)) [16]. Fraction-2 obtained the highest cytotoxic activity and SI (Figure 1b(1,2)). Therefore, 1.92 g of this fraction was fractionated in silica gel-60 with petroleum ether–acetone 8:2 as isocratic mobile phase, resulting in three final sub-fractions (SFA, SFB, SFC). Then, the SFB sub-fraction obtained the highest cytotoxic activity (Figure 1c(1,2)). However, SFB lost some of its cytotoxic activity traits throughout fraction-2 fractionation; nevertheless, SFB presented the highest SI of all sub-fractions (Figure 1c(2)), which is a highly desired characteristic [14,16], pointing to this sub-fraction as the most suitable fraction to be studied further as a potential anticancer phytopharmaceutical. Owing to the nature of the bio-guided study, SFB was later fractionated. 

Likewise, Da costa et al. [17] reported that the fresh sap of the xylem of *H. courbaril* exhibited a higher cytotoxic effect, with an IC_50_ of 109 µg/mL; conversely, the majority compound, Fisetin, had an IC_50_ of 158 µg/mL on the murine embryo fibroblast cell line, Balb/c-3T3-A31 [17], showing a similar behavior to our study, when the cytotoxic activity decreased as the fractionations were carried out in the study. This is mainly because the compounds could be interacting synergistically within the fractions, and when fractionation is carried out, this synergistic potential can be excluded, or perhaps the active components are in fractions of different polarities, resulting in undetectable amounts of these components present in the fraction studied [18]. Subsequently, 1.23 g of the SFB sub-fraction was fractionated in reverse phase column chromatography (RP18) with a gradient of elution starting with chloroform, followed by mixtures of chloroform–hexane 9:1, then, chloroform–hexane 7:3, and so on. Six final sub-fractions were obtained (SFB.1 to SFB.6), which were evaluated through the MTT assay and SI. SFB.3 presented the highest anti-cancer potential (Figure 1d(1,2)).

### 2.2. Identification of the Active Compound by Gas Chromatography-Mass Spectrometry (GC/MS) 

The secondary metabolites were identified from the SFB.3 sub-fraction by GC/MS (Table 1). The identification criteria were based on the percentage of coincidence (≥70%) and the 2.5% abundance limit. OXC was found with 24.95% of relative abundance within the SFB.3 sub-fraction. The SFB.3 sub-fraction was fractioned over silica gel (10× *g*) and chloroform–hexane 9:1 as a solvent to obtain white crystals after crystallization with a melting point of 61.5 °C and UV-VIS l max of 242 nm. The GC/MS for crystals showed a fragmentation pattern with a molecular ion peak at *m*/*z* 220.05 (6.57%), and fragments in *m*/*z* 79.05 (100%), *m*/*z* 93.05 (71.69%), and *m*/*z* 107.05 (66.27%), with a 98.20% match in the NIST14s mass spectral libraries.

**Figure 2:**
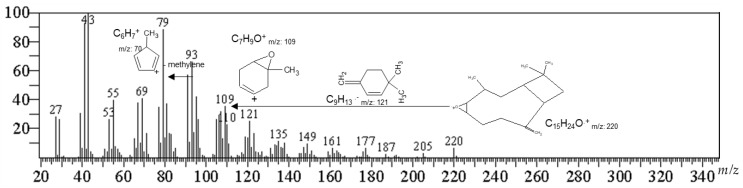
Mass spectrum and partial fragmentation of caryophyllene oxide.

The *m*/*z* 93.05 was derived from the formation of the ion *m*/*z* 136 and the loss of a propene radical (*m*/*z* 43) to afford the ion that confirms the fragmentation of monoterpenes and sesquiterpenes-like molecules. The *m*/*z* 109 diagnostic ion is the *m*/*z* 93 ion but with an additional oxygen molecule, inferring the structure of a sesquiterpene. The base peak ion (*m*/*z* 79) is the loss of a methylene group from the *m*/*z* 93 ions (Figure 2). 

### 2.3. Cytotoxicity and Selectivity Index for OXC

**Figure 3:**
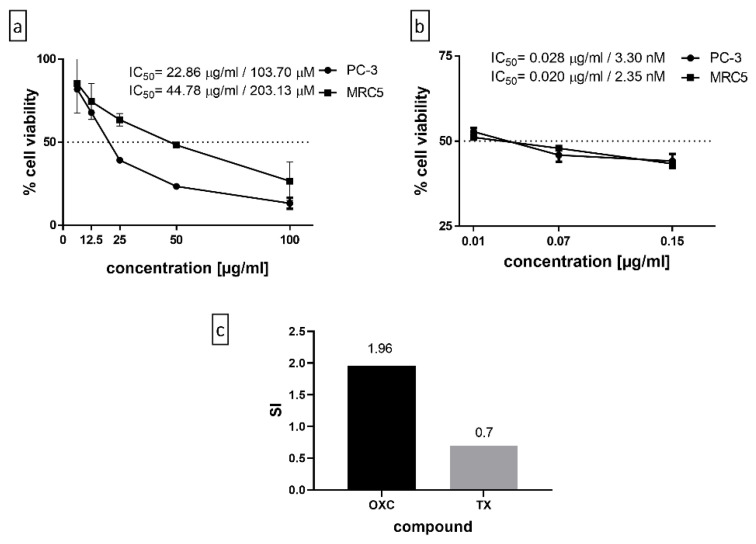
(**a**) The purified compound, OXC, evaluated by the MTT assay, wherein it obtains an IC_50_ of 22.86 µg/mL in PC-3 and 44.78 µg/mL in MRC5 normal cells. (**b**) The positive control, Paclitaxel, 0.028 µg/mL in PC-3 and 0.020 µg/mL in MRC5 normal cells. (**c**) The Selectivity Index of OXC and Paclitaxel, being 1.96 and 0.7, respectively. The experiment was carried out in triplicate with two replicas.

Caryophyllene oxide is a bicyclic sesquiterpene, found as the majority compound in the SFB.3 sub-fraction with a relative abundance of 24.95%. It is mainly known for its insecticidal, antimicrobial, antifungal, analgesic, and antiparasitic properties [19]; alternatively, it also has anti-cancer properties [20,21,22] and can enhance the anti-proliferative efficacy of classical cytostatic agents [20]. The purified compound was evaluated by the MTT assay and SI (Figure 3a,c). In parallel, the OXC was confronted with the positive control, Paclitaxel, a naturally occurring antineoplastic diterpene (*Taxus brevifolia*), now synthetic, used in the treatment of various types of cancers, including prostate, breast, ovarian, and cervical cancer [18] (Figure 3b,c). Regarding the action that OXC could have on PC-3 androgen-independent prostate cancer cells, there are two opposing investigations regarding this action, and there seems to be no clarity. Although Sibanda et al. found that isolated and purified OXC from the essential oil of the *Heteropyxis dehniae* leaf has a 96.75% toxicity at 100 µg/mL when evaluated by the MTS test, the authors do not mention the actual IC_50_, although it is mentioned that the IC_50_ value ranges from 147 to 351 µM (Sibanda et al., 2004), which are higher values than the IC_50_ obtained in this work by the MTT test at 48 h of treatment (103.7 µM). Contrariwise, in another investigation in which the cell viability was measured employing the MTT assay, the results were ambiguous, concluding that PC-3 cell proliferation is suppressed in a time-dependent manner by OXC. It can only be assumed that their IC_50_ is the concentration used for the tests subsequently used during the study, 50 µM. Some of these results are slightly implausible since the authors allege that OXC induces the expression of p53 in a concentration-dependent manner, even suggesting the presence of p53 protein in untreated cells [22], which cannot be true since it is known that PC-3 is a null-p53 cell line [7]. The apoptosis carried out in their investigation could be mediated by p53, thus affecting the cytotoxicity test results.

### 2.4. OXC Exert Morphological Changes Leading to the Emergence of Apoptotic Cells 

**Figure 4:**
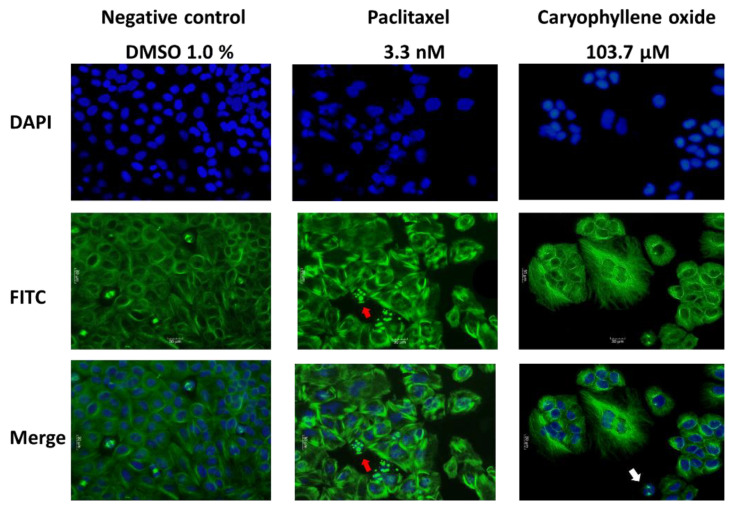
Morphological changes in PC-3 after 24 h of OXC and Paclitaxel exposure. Immunofluorescence micrographs showing the nuclear (in blue) and microtubular (in green) effects in PC-3 cancer cells. Paclitaxel-treated cells displayed a moderate destabilization of the microtubules, an increase in cell size, the appearance of some apoptotic bodies (marked with a red arrow) that were not observed in the negative control cells treated with 1% DMSO, and the formation of abnormal nuclear morphologies. Otherwise, cells treated with OXC were visibly affected at the microtubules, considerably increasing the size of the cells, presenting cellular clusters, variable nuclear morphologies, and leading to the emergence of apoptotic cells (white arrow).

Morphological analysis was performed to examine cellular damages that may be generated after treatments by immunofluorescence microscopy. Paclitaxel-treated cells were destabilized moderately at the microtubules, increasing cell size, with some apoptotic bodies appearing (Figure 4, red arrow) that were not observed in the negative control cells exposed to 1% DMSO, and forming abnormal nuclear morphologies. The effect of OXC was more evident, due to a stronger microtubule destabilization, the dense and compact damage around the nuclei, which were undergoing abnormal formations. Additionally, the cells increased in size, presenting cellular clusters and the appearance of some apoptotic bodies (Figure 4, white arrow). Wang et al. demonstrated that PC-3 cells exposed to Paclitaxel have an abnormal network of microtubules around the nucleus 4–8 h after treatment, which induces the formation of multipolar spindles. However, these cells continue the cycle, even up to G2/M, forming a defective spindle, which then causes the cycle to stop [23].

### 2.5. Caryophyllene Oxide Induces Early and Late Apoptosis

**Figure 5:**
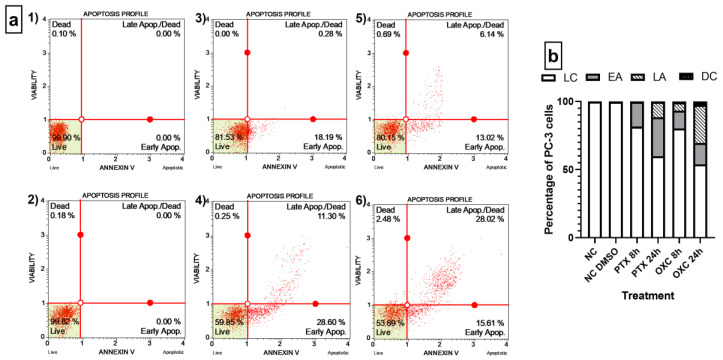
Quantitative detection of Annexin V/7-AAD on PC-3 cells. (**a**) Dot plots of OXC and Paclitaxel treatments in the Annexin V/7-AAD assay. (1) Negative control, cells without any treatment. (2) Negative control, cells exposed to 1% DMSO. (3) Cells exposed to Paclitaxel for 8 h. (4) Cells exposed to Paclitaxel for 24 h. (5) Cells exposed to OXC for 8 h. (6) Cells exposed to OXC for 24 h. (**b**) Bar chart representing the distribution of the PC-3 cells detected by Annexin V/7-AAD following OXC and Paclitaxel exposure. Cells located in the lower right stained with Annexin V were defined as early apoptotic (EA), and Annexin V and 7-AAD double-stained cells were defined as late apoptotic (LA), located in the upper right. In the lower left are cells negative to both dyes, defined as live cells (LC), and cells in the upper left were stained only with 7-AAD, and correspond to non-apoptotic dead cells or nuclear debris.

Morphological changes in the cytoskeleton by binding of Paclitaxel to tubulin polymer prevent its disassembly, thus blocking the cell in the G2/M phase and leading to apoptosis [23], such as was observed in Figure 4 also by OXC in PC-3 cells. To prove whether OXC induces apoptosis, the quantitative analyses of live cells, early and late apoptosis, and cell death were carried out using the Muse^®^ Annexin V and Dead Cell assay. Both compounds, the OXC and the positive control Paclitaxel, induced the early apoptotic pathway, where molecules of phosphatidylserine (PS) translocate to the outer surface of the cytoplasmatic membrane and Annexin V can bind to them; however, after 24 h of treatment, when cells were treated with OXC, the early apoptotic population increased from 13.02% (at 8 h) to 15.61% and the late apoptosis population from 6.14% to 28.02%. Furthermore, OXC caused late apoptosis in a higher percentage of cells than Paclitaxel at 24 h, detected by binding of both Annexin V to PS and 7-amino-actinomycin (7-AAD) to nucleic acids when the membrane integrity is breached [24,25]. After 24 h of OXC treatment, the percentage of total apoptotic cells increased from 19.16% to 43.81%. Conversely, when the cells were exposed to Paclitaxel, the percentage increased from 18.47% to 39.9% (Figure 5a,b). The results demonstrated that PC-3 cells were less resistant to apoptosis induction by OXC than by Paclitaxel. 

### 2.6. Loss of Mitochondrial Membrane Potential (Δψm) Induced by OXC in PC-3 Cells 

Destabilization of microtubules can cause the activation of the intrinsic pathway of apoptosis, which includes the release of cytochrome-C from the mitochondria to the cytosol, which in turn implies the permeabilization of the outer mitochondrial membrane as a consequence of changes in the membrane potential, Δψm, and subsequent channel formation [26]. The mitochondrial membrane potential monitoring was used to determine if OXC-induced apoptosis was associated with Δψm disruption in PC-3 cells. 

**Figure 6:**
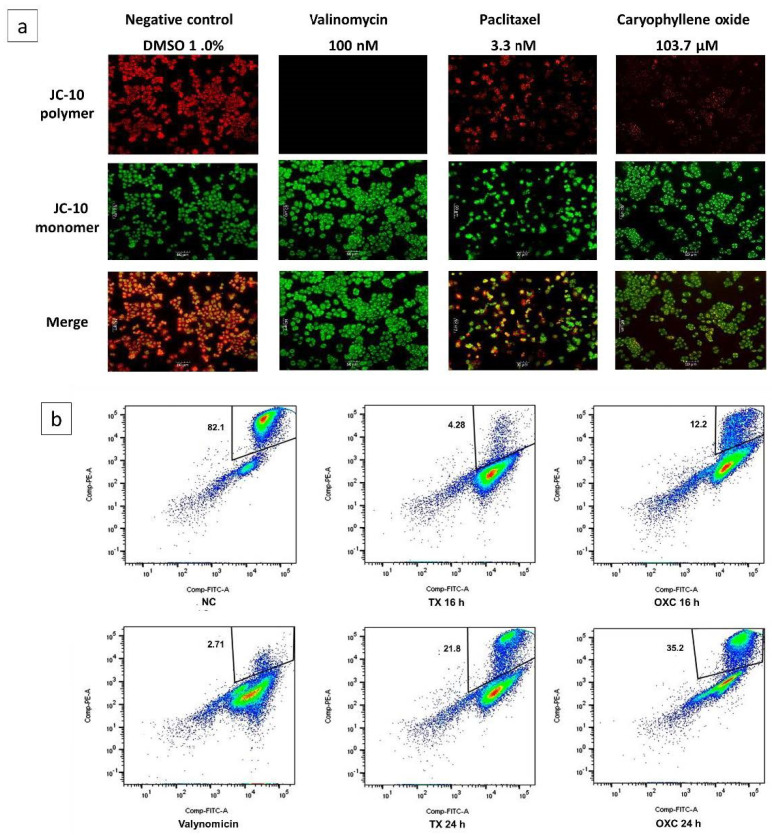
Loss of the membrane potential (Δψm) in PC-3 cells induced by OXC and Paclitaxel (TX). (**a**) Immunofluorescence micrographs show the distribution of JC-10 aggregates in the mitochondrial membrane (in red) or monomers in the cytoplasm (in green) on PC-3 cancer cells after 16 h of incubation at the IC_50_ value, cells treated with 1% DMSO are considered as a negative control (NC). (**b**) Flow cytometry representative density plots evidencing the loss of mitochondrial membrane potential at 16 and 24 h after OXC treatment. Valinomycin was used as a positive control at 100 nM and evaluated at 2 h. The percentage of polarized cells are shown in the box.

Changes were evaluated by fluorescence microscopy and flow cytometry (Figure 6a,b). Initially, PC-3 cells’ responses after being exposed for 16 h to OXC and Paclitaxel at the IC_50_ were studied qualitatively by fluorescence microscopy, wherein cells exposed to OXC exhibit mainly green fluorescence and little red fluoresce, similar to the fluorescence exhibited by the assay positive control, valinomycin (Figure 6a). In healthy cells, the lipophilic dye JC-10 enters selectively to the mitochondria, where it forms red fluorescent aggregates. Meanwhile, the apoptotic cells with low Δψm JC-10 diffuse out of the mitochondria, where they change to the monomeric form and the cells are stained in green fluorescence [26]. Hence, it suggests that PC-3 cells treated with OXC lose Δψm, and this induces cell death through the mitochondrial-dependent apoptotic pathway [27]. 

When cells were treated with Paclitaxel, the red and green fluorescence alike could be observed. This compound enters a “hyperpolarized” state, probably due to the amount of free tubulin. The alteration of Δψm by Paclitaxel is referred to as “biphasic”. In the initial phase, the cells are depolarized, and later they become a “state of resistance” in which the percentage of depolarization can reach the same level as the untreated cells [26,27,28,29]. Δψm was also evaluated by flow cytometry, and Figure 6b shows the depolarization behavior of the compounds used in this assay. Seemingly, PC-3 behaves differently over the course of the treatment; after both treatments, the polarized state of the cells diminished over time (Figure 6b). When the cells were exposed to OXC for 16 h, they were mostly depolarized (87.8%), however, after 24 h of exposure, the cells decreased their polarized state (64.8%). Cells exposed to Paclitaxel for 16 h were 95.74% polarized, but after 24 h of exposure, they became 78.2% polarized (Figure 6b). These changes in the mitochondrial membrane depolarization may occur in a time-dependent manner, known as the “repolarization” of cells [26,27,28,29]. Similar behavior was observed in the cells treated with the positive control. According to the results, the OXC could act similarly to Paclitaxel, causing a temporary decrease in the Δψm, since hyperpolarization was observed at 24 h.

### 2.7. Apoptotic Proteins’ Expression in PC-3 Cells after Being Exposed for 6, 16, 24, and 48 h to OXC

**Figure 7:**
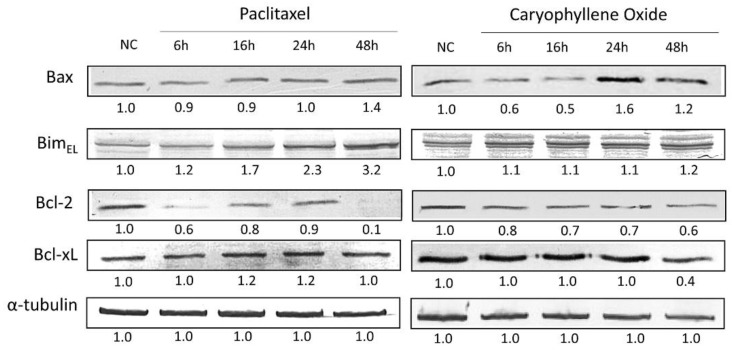
Comparison between the pro-apoptotic and anti-apoptotic protein levels in PC-3 cells treated for up to 48 h with OXC and Paclitaxel. The readouts, defined as the protein levels, were normalized to the readouts of α-tubulin, which are shown below each band. After OXC treatment, the Bax protein expression level decreases until 16 h, then increases at 24 h. Bim protein is maintained without a significant increase until 48 h. Bcl-2 protein was observed to decrease in a time-dependent manner, while Bcl-xL protein remained constant until 24 h, and finally decreased at 48 h. The immunoblot images were semi-quantitated by ImageJ according to the relative densitometric units of each band.

**Figure 8:**
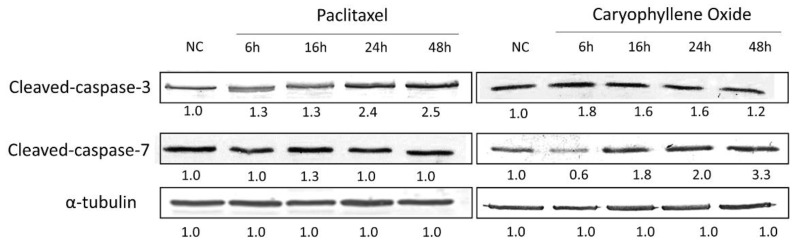
Caspases 3 and 7 activations in PC-3 cells treated for up to 48 h with OXC and the control Paclitaxel. In cells with OXC, the activation of Caspase-3 increases at 6 h and remains until 24 h. Additionally, it leads to an increase in active Caspase-7 in a time-dependent manner. The immunoblot images were semi-quantitated by ImageJ according to the relative densitometric units of each band, and the readouts, defined as the protein complex levels, were normalized to the readouts of α-tubulin, which are shown below each band.

Members of the Bcl-2 family regulate the intrinsic mitochondrial apoptotic pathway for cytochrome-C release and subsequent activation of the caspase cascade [30,31]. Therefore, certain molecular mechanisms involved during apoptosis induction were further examined. The ability of the majority compound to regulate the level of expression of active caspases 3 and 7, in addition to the apoptotic proteins associated with the mitochondria, Bax, Bim, Bcl-2, and Bcl-xL, were evaluated and contrasted with the Paclitaxel known effects. This assay was assessed in PC-3 cells exposed for 6, 16, 24, and 48 h to Paclitaxel and OXC, and changes in the protein levels were measured by Western blot (Figure 7 and Figure 8).

Pro-apoptotic proteins are expected to increase their level of expression during apoptosis. Despite the absence of the p53-specific transactivation function in PC-3 cells, p53 mutants can induce apoptosis to a similar extent to that observed in wt-p53 cell lines. In cells with a mutated p53 phenotype, apoptosis is mediated primarily through the Bax/Bcl-2 relationship [23], and also by Bim protein expression, an essential tumor suppressor for apoptosis induction [32]. Results showed that after OXC treatment, the Bax protein expression level increased at 24 h (Figure 6), and although no important changes were found with the expression of Bim protein during treatment, it could be possible that Bim protein permits Bax release from anti-apoptotic proteins such as Bcl-2, even in those with a constant expression level. Previously, it was suggested that OXC treatment reduces pro-cancer genes/protein expression, while it increases the levels of those with pro-apoptotic properties [20]. In addition, refractory and advanced PCs generally exhibit high levels of Bcl-2 and Bcl-xL anti-apoptotic proteins, contributing to defective apoptosis, which is associated with a poor prognosis, disease progression, and resistance to treatments, which can also contribute to the apoptosis-resistant phenotype in PC-3 cells [23,33]. Conversely, anti-apoptotic protein levels are expected to decrease; however, OXC treatment induced a Bcl-2 protein decrease in a time-dependent manner, but Bcl-xL protein remained constant until 48 h, and with Paclitaxel, that expression was augmented (Figure 6), suggesting that Bcl-xL protein has a protective role in the induction of apoptosis [33].

Caspase activation has been correlated with the apoptosis phase point [34]. With Paclitaxel treatment, the induction of apoptosis through the activation of Caspase-3 is set, even until 48 h (Figure 8), but OXC increases this activation at 6 h, then afterward, it tends to decrease slowly, contrary to results for cleaved-Caspase-7, which increases in a time-dependent manner. Caspase-7 is responsible for the accumulation of ROS production, contributing to the collapse of Δψm, therefore inducing apoptosis and also contributing to the process of cell shedding [35].

### 2.8. Protein–Protein Interactions after Treatment with OXC

**Figure 9:**
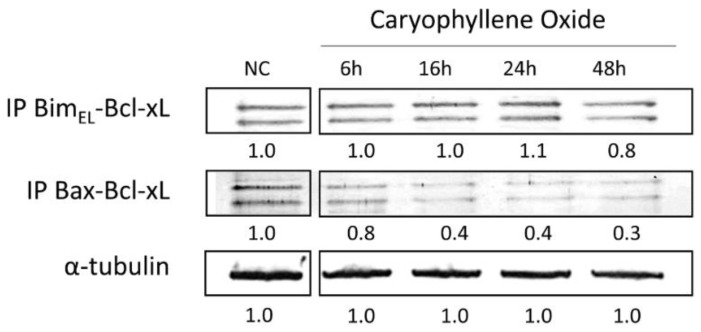
The Bcl-xL/Bax and Bcl-xL/Bim protein complexes’ formation in PC-3 cells exposed to OXC for up to 48 h. The Bcl-xL/Bim protein complex levels remained until 48 h, while the Bcl-xL/Bax complex decreased at 6 h. Protein levels were normalized to the readouts of α-tubulin. The immunoblot images were semi-quantitated by ImageJ according to the relative densitometric units of each band.

Immunoprecipitation analyses showed that the antiapoptotic protein Bcl-xL interacted with the pro-apoptotic proteins Bim and Bax. The Bcl-xL/Bax complex interaction was weaker than Bcl-xL/Bim, because complex levels decreased at the same time as cells were exposed to OXC, in a time-dependent manner (Figure 9). This decrease is consistent with the release of the antiapoptotic complexes to initiate apoptosis through the activation of Bax/Bak [36]. This is a key result since it is known that Bcl-xL protein is a potent anti-apoptotic factor that mediates apoptosis resistance in PC-3 cells and can neutralize the release of cytochrome-C. The heterodimerization of Bcl-xL with Bax/Bak represents an apoptosis blocking mechanism that facilitates tumor progression and resistance to androgens. This interaction was evidenced by Castilla et al. in PC-3 cells at 36 h after being treated with camptothecin, but it was not evident in LNCaP cells with the same treatment [37]. Regarding the Bim/Bcl-xL interaction, Bim antagonizes Bcl-xL to initiate apoptosis, then, a Bim protein shift is necessary so that Bax/Bak activation is carried out, and therefore, the induction of apoptosis, which is known as an indirect activation model [32,33], and this is consistent with the protein interaction between Bcl-xL and Bim (Figure 9).

## 3. Materials and Methods

### 3.1. General Conditions

The *H. coubaril* plant was collected in the metropolitan area of Bucaramanga, Santander, Colombia (7.11° N, 73.11° W), after an ornamental pruning. A specimen was collected and identified at the herbarium of Pontificia Universidad Javeriana and classified with voucher number: HPUJ-30302. Six-hundred and twenty grams of the leaves of the plant were subjected to Soxhlet extraction with hexane (three days), followed by dichloromethane (three days) and then ethanol (eight days), thereby obtaining three extracts with different polarities [38]. Stock solutions of the extracts, fractions, and the isolated compound caryophyllene oxide (OXC) were prepared at a concentration of 10 mg/mL, and of the positive control Paclitaxel (Cayman Chemical, Ann Arbor, MI, USA) at a concentration of 10 μg/mL in dimethylsulfoxide (DMSO) (Sigma-Aldrich, St. Louis, MO, USA). These stock solutions were used for all tests. Cultured cells were maintained as follows: prostate adenocarcinoma (PC-3) (ATCC^®^, Manassas, VA, USA, CRL1435™) and lung fibroblasts (MRC-5) (ATCC^®^, Manassas, VA, USA, CCL-171™) in supplemented EMEM (Lonza, CH, Walkersville, MD, USA) medium with 10% (*v*/*v*) Fetal Bovine Serum (Biowest, Riverside, MO, USA), 2 mM L-glutamine, and 5000 IU/mL penicillin and 5 mg/mL streptomycin (Lonza, CH, Walkersville, MD, USA). Incubation was performed at 37 °C and 5% CO_2_. 

### 3.2. Cell Viability Assay

To calculate the cytotoxic activity and Selectivity Index of substances from *H. courbaryl*, approximately 7000 PC-3 cells/well were seeded in a 96-well plate. Extracts, fractions, and active molecules treatment concentrations ranged between 6.25 and 150 µg/mL, and 0.01–0.15 µg/mL for Paclitaxel, followed by 48 h of incubation. Then, 100 µL of 0.5 mg/mL of MTT solution (Sigma-Aldrich, St. Louis, MO, USA) was added and incubated for 4 h. Formazan crystals were dissolved with 100 µL of DMSO. The results were determined by the optical density determined by the absorbance at 570 nm [5,39]. Estimation of the half-maximal inhibitory concentration (IC_50_) was performed using non-linear regression from plotting cell survival (%) versus treatment concentration (µg/mL). The Selectivity Index (SI), which represents IC_50_ for normal cell line/IC_50_ for cancer cells after 48 h of treatment, was applied as exclusion criteria for fractions [40]. The IC_50_ values were submitted to a one-way analysis of variance (ANOVA) with post-hoc Tukey and Scheffé tests using SPSS. Tests were considered statistically significant at *p* < 0.05. All the experiments were performed in triplicate with at least two independent replicates, and results are presented as mean ± SEM.

### 3.3. Detection of Morphological Changes by Immunofluorescence

About 7000 PC-3 cells/well were seeded in a 96-well plate, then treated at the IC_50_ previously determined. After 24 h of incubation, cells were fixed in methanol (−20 °C), then permeabilized with a 1:1 Phosphate-Buffered Saline (PBS)/acetone mixture (−20 °C) for 20 s. To evaluate the integrity of the microtubules, the monoclonal Anti-α-Tubulin-T9026 (Sigma-Aldrich, St. Louis, MO, USA) and the goat anti-mouse Alexa Fluor 488 (Molecular Probes, Eugene, OR, USA) antibodies were used, both diluted in 5% BSA/TTBS (*w*/*v*) blocking solution (bovine serum albumin/Tris HCl pH 7.5 NaCl, and Tween 20). DNA staining was performed with 1.0 mg/mL of DAPI (Invitrogen, Waltham, MA, USA). Fluorescence was monitored using an epifluorescence microscope (Motic AE31, Schertz, TX, USA), and the images were captured with MoticCamPro 282A and analyzed using Motic Image plus 2.0 software [5,23,39].

### 3.4. Secondary Metabolites’ Identification 

The fractionation of the hexane extract was performed by CC with an isocratic mobile phase of petroleum acetone–ether 8:2 (Merck, ACS, Reag. Ph Eur, DE, Darmstadt, Germany). The purification was performed over 10 g of silica gel, and chloroform–hexane 9:1 was used as a solvent until crystals were obtained. The cytotoxicity of these fractions and compounds was performed by the MTT assay to later determine the SI [40]. Qualitative identification of secondary metabolites present in total fractions and sub-fractions with anti-proliferative potential according to the IC_50_ and SI were carried out by gas chromatography, using a Shimadzu GC 2010 brand instrument coupled to a TQ8040 triple quadrupole selective mass detector (Shimadzu Scientific Instruments, Columbia, MD, USA), with electronic impact ionization at 70 eV, in split mode (1:50), and a GC/MS Real-Time Analysis V. 4.45 data system. An SH-Rxi-5ms column (Shimadzu, Columbia, MD, USA) with the following characteristics: 30 m × 0.25 mm × 0.25 μm, with oven programming: 60 °C (5 min) at 5 °C/min, up to 150 °C (3 min), at 4 °C/min, up to 310 °C for 5 min, was used. The total running time was 65.5 min. The injector temperature was 250 °C using helium 5.0 (Cryogas, 99.9990% purity, Col) as a carrier gas at 1.56 mL/min. The temperatures of the ion source and the interfaces were 230 and 250 °C, respectively. The identification criteria were based on the percentage of coincidence (≥70%) of the compounds obtained in comparison with the bases of NIST14 and NIST14s compounds (NIST, Gaithersburg, MD, USA) as mass spectral libraries.

### 3.5. The Muse Annexin V and Dead Cell Assay

The quantitative analysis of live cells, early and late apoptosis, and cell death was analyzed using the Muse^®^ Annexin V and Dead Cell Reagent (Luminex Corporation, Austin, TX, USA) on the Guava^®^ Muse^®^ Cell Analyzer. Following PC3 cell culture, cells were treated with Paclitaxel and OXC at the corresponding IC_50_ for 8 and 24 h. Positive and negative controls were also cultured. Cellular samples at a final concentration of 1 × 10^4^ cells/mL were prepared, and 100 µL of Muse^®^ Annexin V and Dead Cell Reagent were added to each tube containing 100 µL of the cellular suspension. Cell suspension with the reagent was incubated for 20 min at room temperature in the dark. Later, the samples were measured in the cell analyzer (Luminex Corporation, Austin, TX, USA) [24].

### 3.6. Analysis of the Mitochondrial Membrane Potential (Δψm)

The changes in the mitochondrial membrane potential were analyzed using the JC-10 probe (ultra, pure, Enzo Life Science, Farmingdale, NY, USA). The JC-10 probe was previously dissolved in DMSO (5 mg/mL) to obtain a stock solution; later, a fresh working solution (20 µg/mL) was prepared in a culture medium at 37 °C. For fluorescence analysis, approximately 7000 cells/well were seeded in a 96-well plate. OXC and Paclitaxel treatments were administered at the IC_50_ and then incubated for 16 h. On the day of the assay, a stock of the positive control for this assay, Valinomycin (Cayman Chemical, Ann Arbor, MI, USA) (100 nM), was prepared, administrated, and incubated for 2 h at 37 °C, 5% CO_2_. Then, cells were washed with sterile 1× PBS, and 50 µL of the staining solution (5 mg/mL) was added and incubated for 30 min before observing fluorescence using an epifluorescence microscope (Motic AE31, Schertz, TX, USA). The images were captured with MoticCamPro 282A and analyzed using Motic Image plus 2.0 software [23,25,26]. For flow cytometry, approximately 200,000 cells/well were seeded in a 12-well plate. Cells were treated with OXC and Paclitaxel for 16 and 24 h. On the day of the assay, Valinomycin was administered at 100 nM for 2 h before reading the test. Afterward, the cells were washed with sterile 1× PBS and subsequently removed and centrifuged, then 50 µL of the working solution was added and incubated for 30 min. The cells were centrifuged again, and PBS was added to perform the cytometer reading (BD-FACSAria cytometer, San Jose, CA, USA) [26]. 

### 3.7. Detection of Apoptotic Proteins by SDS-PAGE and Western Blot

Protein extraction was carried out with approximately 2 × 10^6^ cells treated for 6, 16, 24, and 48 h with OXC or Paclitaxel at the corresponding IC_50_ using a lysis buffer (20 mM Tris HCl pH 8.0, 137 mM NaCl, 10% glycerol, 1% NP-40, and 10 mM EDTA). The total protein concentration was determined by the Bicinchoninic Acid (BCA) Protein Assay Kit (Pierce), and 25 µg of protein from each sample were subjected to 11% SDS-PAGE polyacrylamide gel electrophoresis using a mini-gel system (mini-Protean II; Bio-Rad Laboratories). Proteins were blotted using polyvinylidene fluoride membranes (PVDF). The transferred membranes were blocked for 1 h with 5% BSA/TTBS (*w*/*v*), followed by overnight incubation at 4 °C with the primary antibodies: anti-Bim, anti-Bax, anti-Bcl-2, anti-Bcl-xL, anti-Caspase-7 (GeneTex, Irvine, CA, USA), or anti-Caspase-3 (Thermo Fisher Scientific, Waltham, MA, USA), and anti-α-tubulin (Merck Group, DE, Darmstadt, Germany) was used as a loading control. On the next day, the membranes were incubated with the corresponding secondary antibody, either anti-Mouse IgG or anti-Rabbit IgG (Merck Group, DE, Darmstadt, Germany), for 1 h at room temperature. Bands were visualized on PVDF membranes using the 3,3′-diaminobenzidine tetrahydrochloride (DAB) Substrate Kit detection method (Pierce). ImageJ software was used to semi-quantify and compare band density [5].

### 3.8. Bcl-xL Protein Complex Formation by Co-Immunoprecipitation

Of the total protein, 150 μg was mixed with 2 μg of the anti-Bcl-xL antibody (Genetex, Irvine, CA, USA) and buffer (50 mM Tris HCl pH 7.4, 0.5 mM EDTA, 1% NP40, 50 nM NaF, 0.5 mM PMSF, and protease inhibitors cocktail (1:100) (Pierce, WA, USA)) [30]. Samples were incubated at 4 °C for 4 h. Later, the samples were incubated with the resin (Protein G) (Thermo-Fisher Scientific, Waltham, MA, USA) at 4 °C overnight, and then were washed with the wash buffer (50 mM Tris HCl pH 7.4, 250 mM NaCl, 0.5 mM EDTA, 1% NP40, 50 nM NaF, 0.5 mM PMSF, and protease inhibitors cocktail (1:100) (Pierce, WA, USA)); subsequently, samples were denatured with 1× loading buffer and boiled for 5 min (90 °C) [5,26]. The detection of Bim and Bax proteins linked to Bcl-xL protein was conducted using the previously described protocols for electrophoresis and Western blot.

## 4. Conclusions

OXC was identified as the most active compound from the bio-guided fractionation of the leaf hexane total extract of *Hymenaea courbaril* L. It was recognized as a potential anti-proliferative agent for the treatment of androgen-independent prostate PC-3 cancer cells. Cells exhibited morphological changes after treatment. The increased protein levels of Bax and Bim, as well as the reduction in Bcl-2 and Bcl-xL protein levels and the decrease in the Δψm after OXC treatment, are typical behaviors during induction of apoptosis. OXC induced early and late apoptosis dependent on both active Caspase-3 and in a high level of Caspase-7. *H. courbaril* may provide compounds with potential against prostate cancer.

## Figures and Tables

**Table 1:** Secondary metabolites found in the sub-fraction SFB.3.

Compound	t_R_ (Min)	Base Peak	Diagnostic Ions *m*/*z* (%)	Area % (Abundance)
(1R,7S E)-7-Isopropyl-4,10-DiMethylenecyClodec-5-Enol	27.544	105.05 (100)	91.05 (93.75), 131.1 (91.68), 93.05 (69.45), 159.1 (63.17), 55.05 (58.92)	12.14
	29.346	91.05 (100)	93.05 (81.21), 79.05 (80.58), 105.1 (67.12), 107.1 (65.48), 81.1 (59.08)	
	29.976	159.1 (100)	91.05 (68.01), 93.05 (62.62), 105.1 (62.43), 81.1 (49.6), 131.1 (48.79)	
Caryophyllene Oxide	30.949	93.1 (100)	55.05 (76.72), 91.1 (68.76), 107.1 (67.28), 67.05 (64.59), 79.05 (64.22)	24.95
	31.719	79.05 (100)	91.05 (80.93), 93.05 (71.69), 95.05 (67.83), 67.05 (67.8), 107.05 (66.27)	
	33.313	79.1 (100)	55.05 (98.23), 81.05 (97.82), 91.05 (97.07), 109.05 (96.05), 95.1 (95.3)	
γ-Sitosterol	63.317	105.1 (100)	81.1 (92.99), 107.1 (91.59), 55.05 (90.65), 95.1 (86.66), 145.1 (83.99)	23.42

## Data Availability

There are no publicly archived datasets analyzed or generated during the study.

## Sample Availability

Samples of the compounds are available from the authors.
